# Supplementary material for: Odor-evoked inhibition of olfactory sensory neurons drives olfactory perception in Drosophila
Source: Nat Commun. 2017 Nov 7;8:1357. doi: 10.1038/s41467-017-01185-0 (PMC5676773; doi:10.1038/s41467-017-01185-0)
Supplement: Supplementary file 1 — Supplementary Information [file 41467_2017_1185_MOESM1_ESM.pdf]

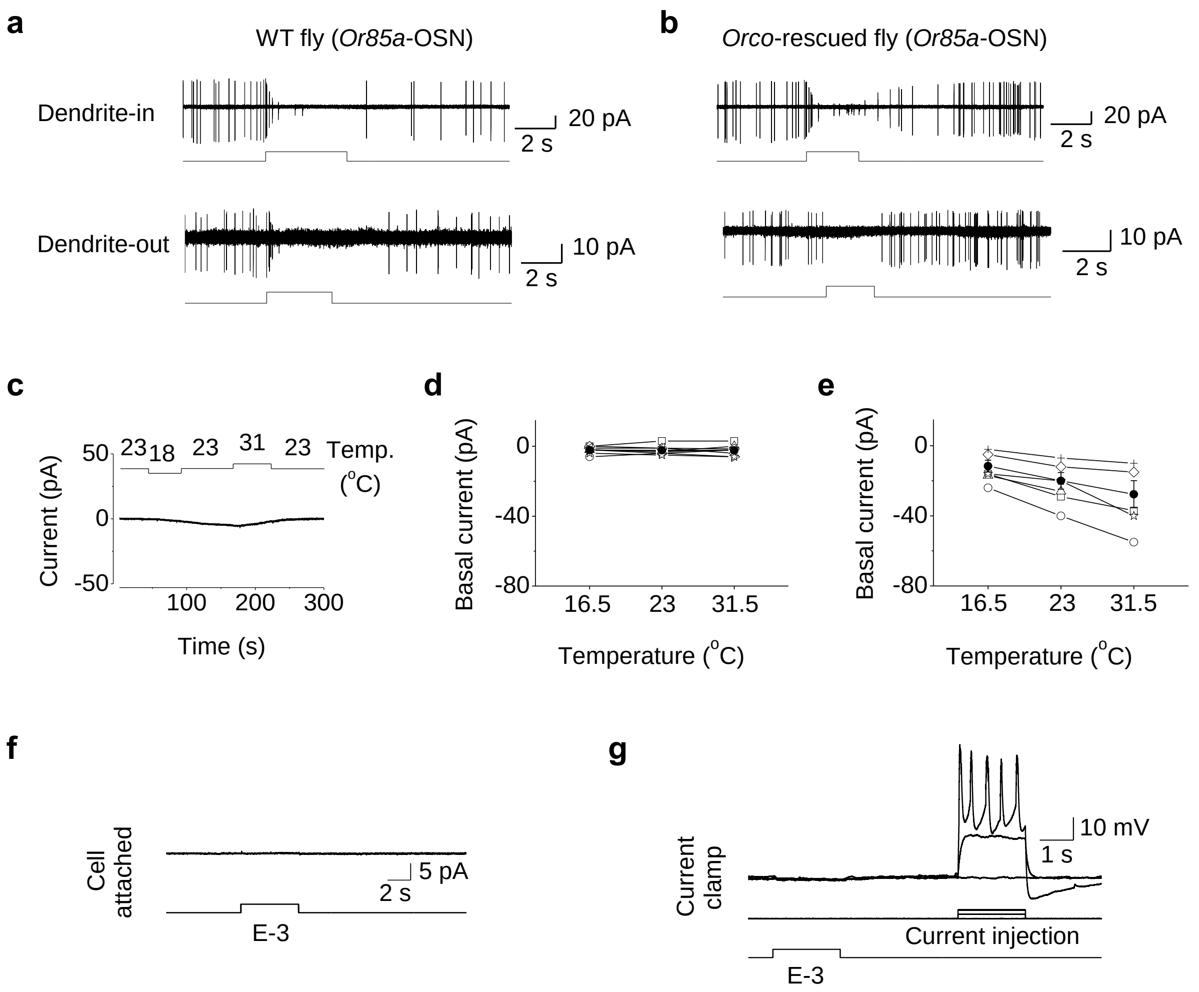

Supplementary Figure 1

Direct odor inhibition, temperature and *Orco* dependence of basal receptor current in *Or85a*-OSNs

(**a**) Cell-attached recordings from *Or85a*-OSNs in WT flies. Top, recording from an *Or85a*-OSN with its sensory dendrite remaining intact in the sensillum (Dendrite-in)[25]. Bottom, recording from an isolated *Or85a*-OSN with its dendrite pulled out from the sensillum socket and directly exposed to the bath perfusion (Dendrite-out) [25], which eliminates ephaptic inhibition from the neighboring OSN dendrites in the same sensillum[26]. Timing of the odor stimulation is indicated at the bottom. Odor concentration: 10 mM acetophenone. (**b**) Cell-attached recordings from *OR85a*-OSNs in the flies with *Orco* restored in *Orco*$^{-/-}$ background. Top, recording from an *Or85a*-OSN in the Dendrite-in configuration. Bottom, recording from an *Or85a*-OSN in the Dendrite-out configuration. Odor concentration: 10 mM acetophenone. (**c**) No change in basal current by temperature in *Or85a*-OSNs of *Orco*$^{-/-}$ flies. Temperature changes are indicated above the recording trace. (**d**) Collective data for the basal current at different temperatures in *Or85a*-OSNs of *Orco*$^{-/-}$ flies, with average indicated in black dots. (**e**) The collective data

of temperature-dependence of the basal current in *Or85a*-OSNs of WT flies, with average indicated in black dots. (**f**) Loss of spontaneous activity and odor-evoked responses in *Or85a*-OSNs of *Orco*$^{-/-}$ flies. Under the cell-attached configuration, strong odor stimulation with E-3 (10 mM, 5 s) did not trigger any action-potential firing. (**g**) The dependence of odor-evoked depolarization on the presence of *Orco*. Under the current clamp, odor stimulation as in (**a**) did not induce any depolarization in the perforated-patch-clamped *Or85a*-OSNs of the *Orco*$^{-/-}$ flies. Injections of inward currents (3 and 5 pA; 5 s) depolarizes the OSN and triggers bursts of firing, indicating that the OSN is electrically excitable.

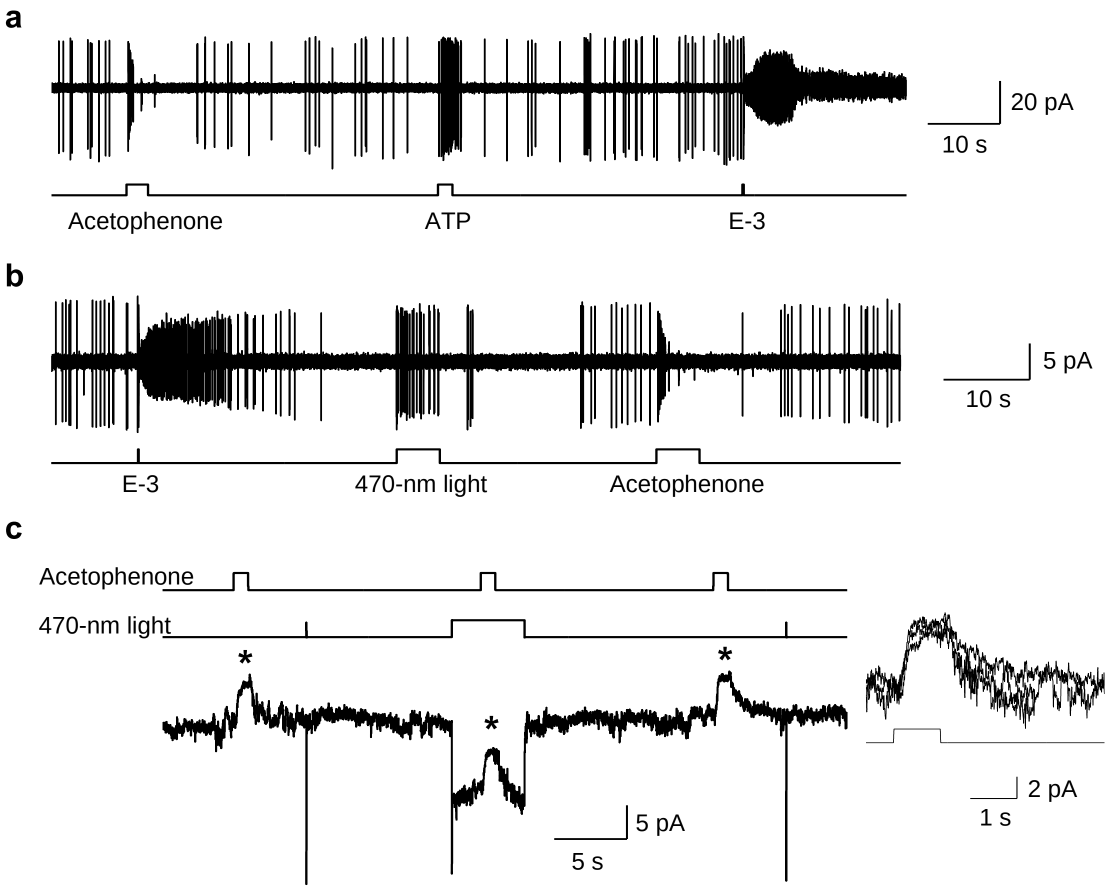

Supplementary Figure 2

ATP and light-induced excitatory responses in *Or85a*-OSNs

(**a**) Under the cell-attached configuration, *Or85a*-OSNs expressing the ATP-gated P2X$_2$ cation channel were stimulated by acetophenone (20 mM, 3 s), ATP (1 mM, 2 s), and E-3 (1 mM, 35 ms). (**b**) Spike firing induced by both odor and light in *Or85a*-OSNs. Under the cell-attached configuration, the OSN expressing ChR2 was stimulated by E-3 (1 mM, 35 ms), acetophenone (20 mM, 5 s), and 470 nm light (5 s). The timing of odor and light stimulation is indicated at the bottom. (**c**) Acetophenone inhibited the basal current but not the ChR2-induced current. Inset, overlap of the acetophenone-induced outward currents in the absence or presence of light-induced inward current. The timing of the application of acetophenone and 470-nm light (10 ms) is indicated on the top.

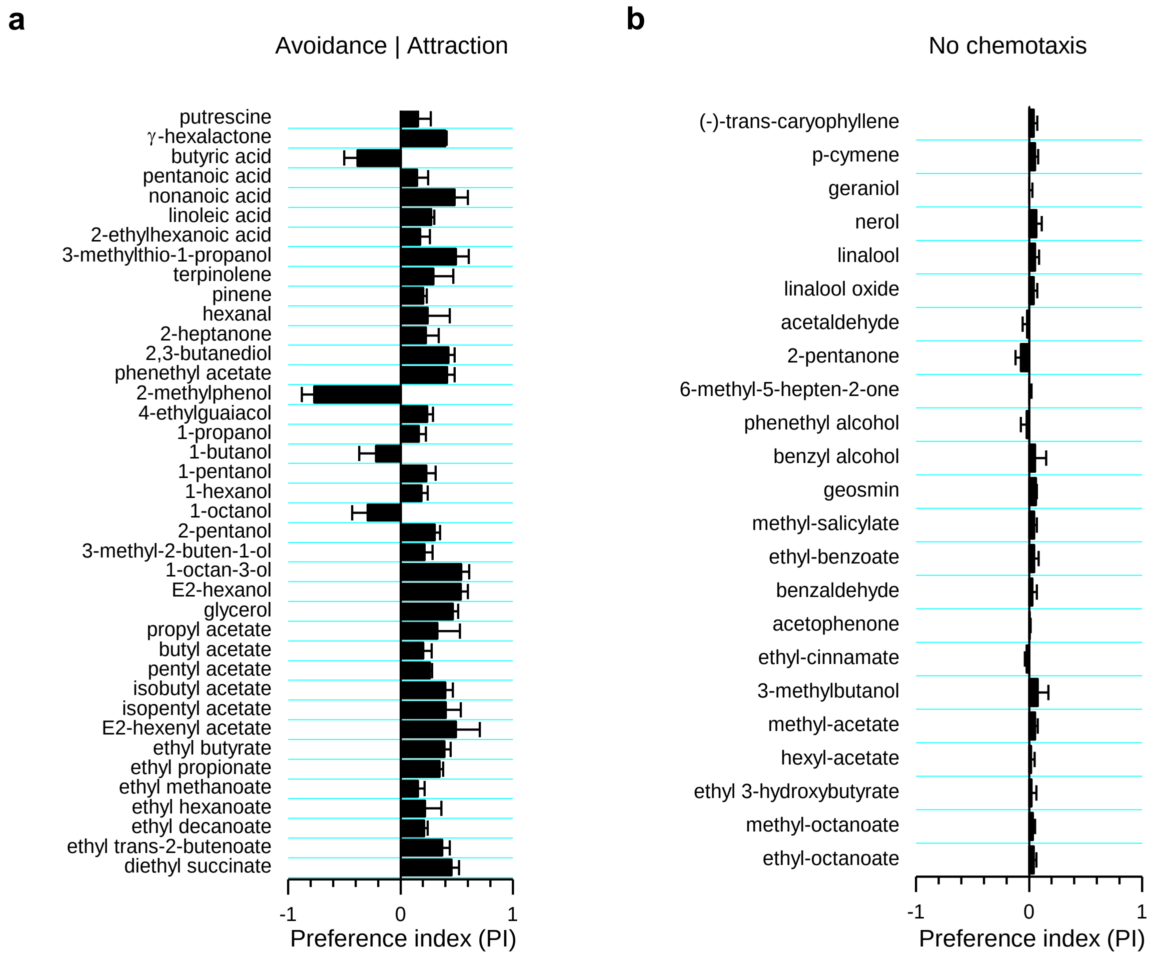

Supplementary Figure 3

Chemotaxis of *Orco*$^{-/-}$ flies to odors

(**a**) Avoidance and attraction by different odors, which are probably mediated by other chemoreceptors, such as ionotropic receptors and gustatory receptors. Odors as indicated (dissolved in mineral oil or water). (**b**) Odors did not trigger obvious chemotaxis in *Orco*$^{-/-}$ flies.

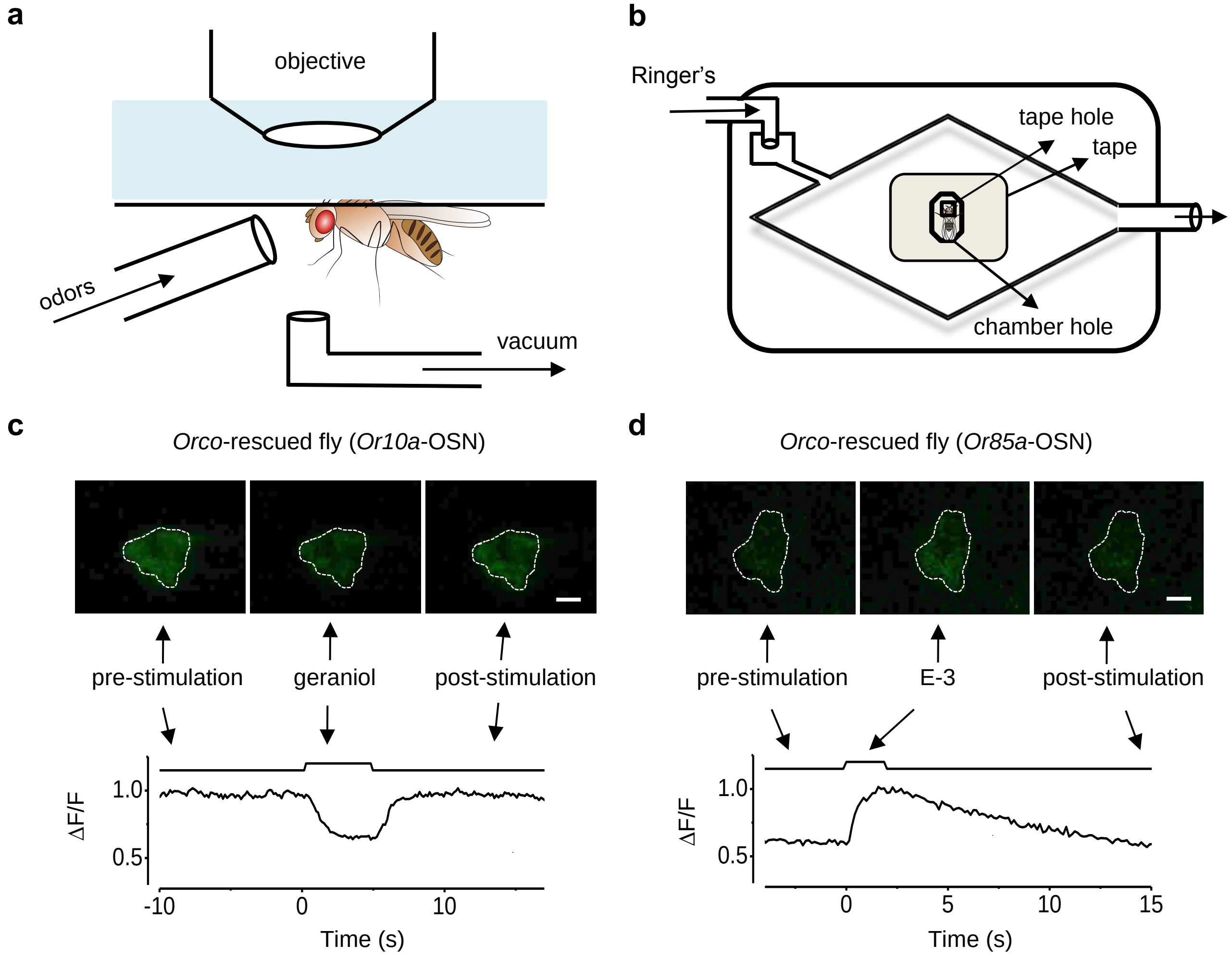

Supplementary Figure 4

Calcium imaging of the antennal lobe in a live fly

(**a**) Schematic of the calcium imaging and odor stimulation. (**b**) Schematic of the recording chamber. (**c**) Odor-evoked inhibitory responses. (**d**) Odor-evoked excitatory responses. Scale bar: 10 μm.

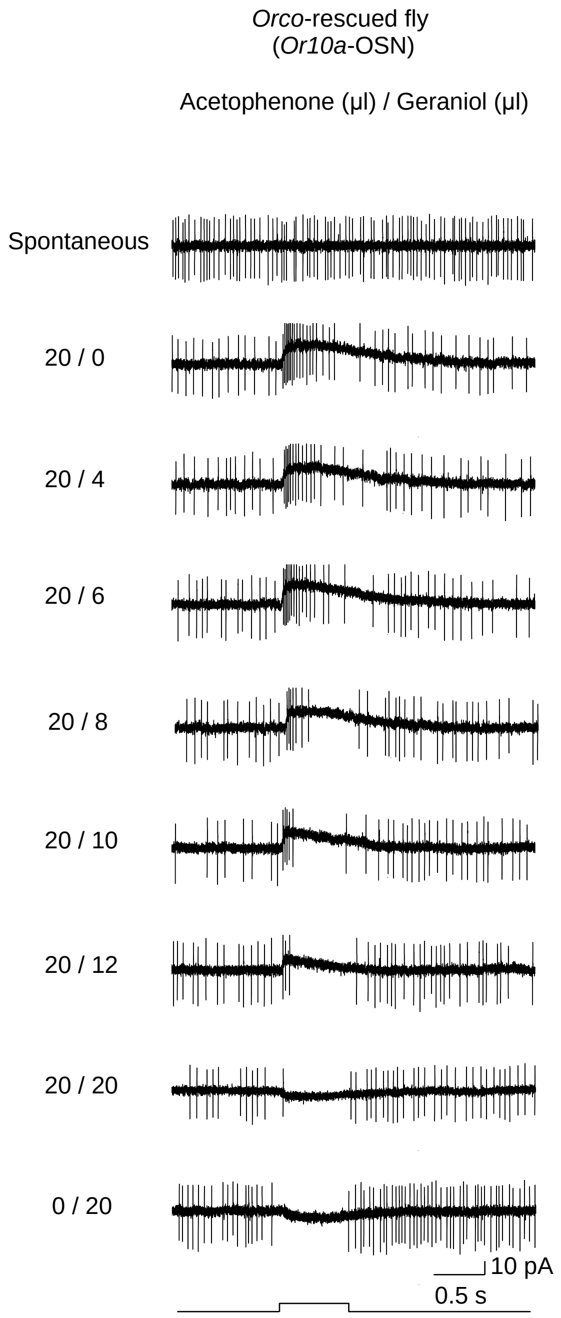

Supplementary Figure 5

Spike firing of *Or10a*-OSNs in response to odor mixtures

Single-sensillum recordings were performed on ab1 sensilla of *Orco*$^{-/-}$ flies with *Orco* restored to *Or10a*-OSN. Odor stimulations were mixtures of acetophenone (10^{-2} dilution) and geraniol (10^{-2} dilution) as indicated. Note: the odor stimulation would be further diluted before reaching the recorded OSNs; these results thus do not directly match the corresponding behavioral conditions.

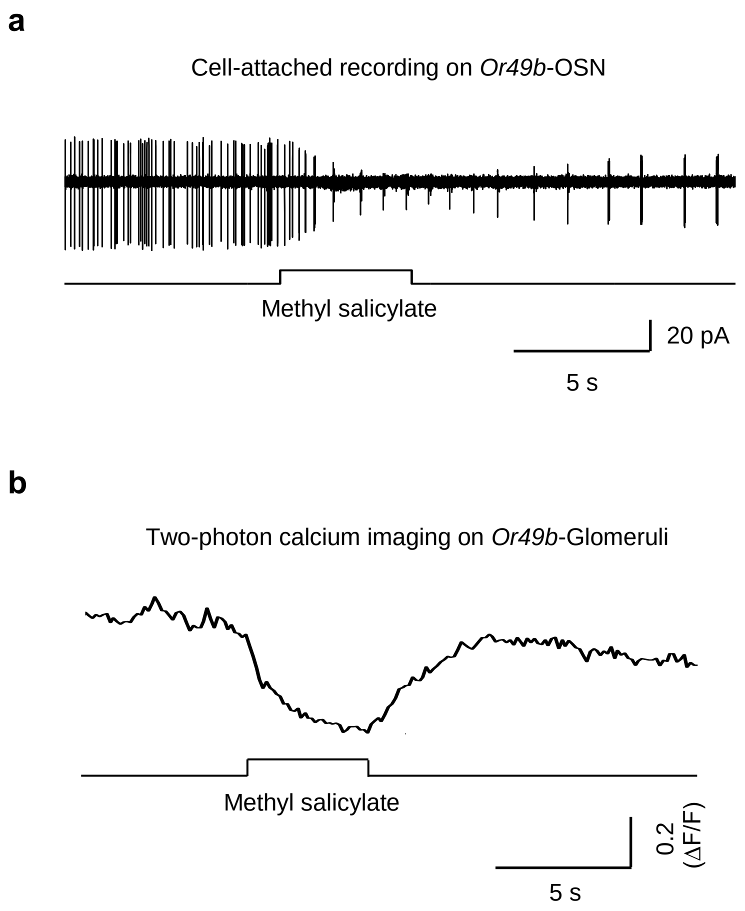

Supplementary Figure 6

Methyl salicylate-evoked inhibitory responses in *Or49b*-OSNs

(**a**) Cell-attached recordings on *Or49b*-OSN. Odor stimulation: 3 mM methyl salicylate, applied for 5 s. (**b**) Two-photon calcium imaging on the glomeruli labeled by GCaMP6m expressing in *Or49b*-OSNs.

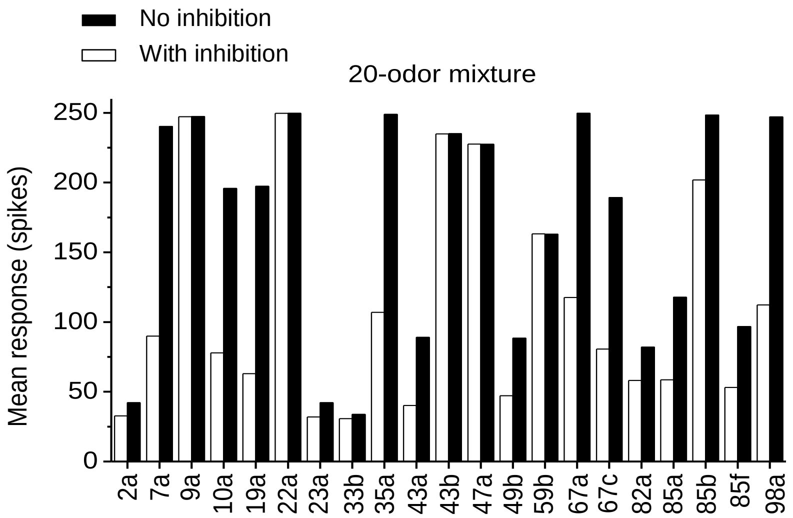

Supplementary Figure 7

Reducing response saturation by inhibition

The average activity (spiking rate) of each OR/OSN in response to odor mixtures containing 20 odors. The random-sampling method with a total of 100,000 odor mixtures was used. The inclusion of inhibition reduces the response saturation of many ORs/OSNs.

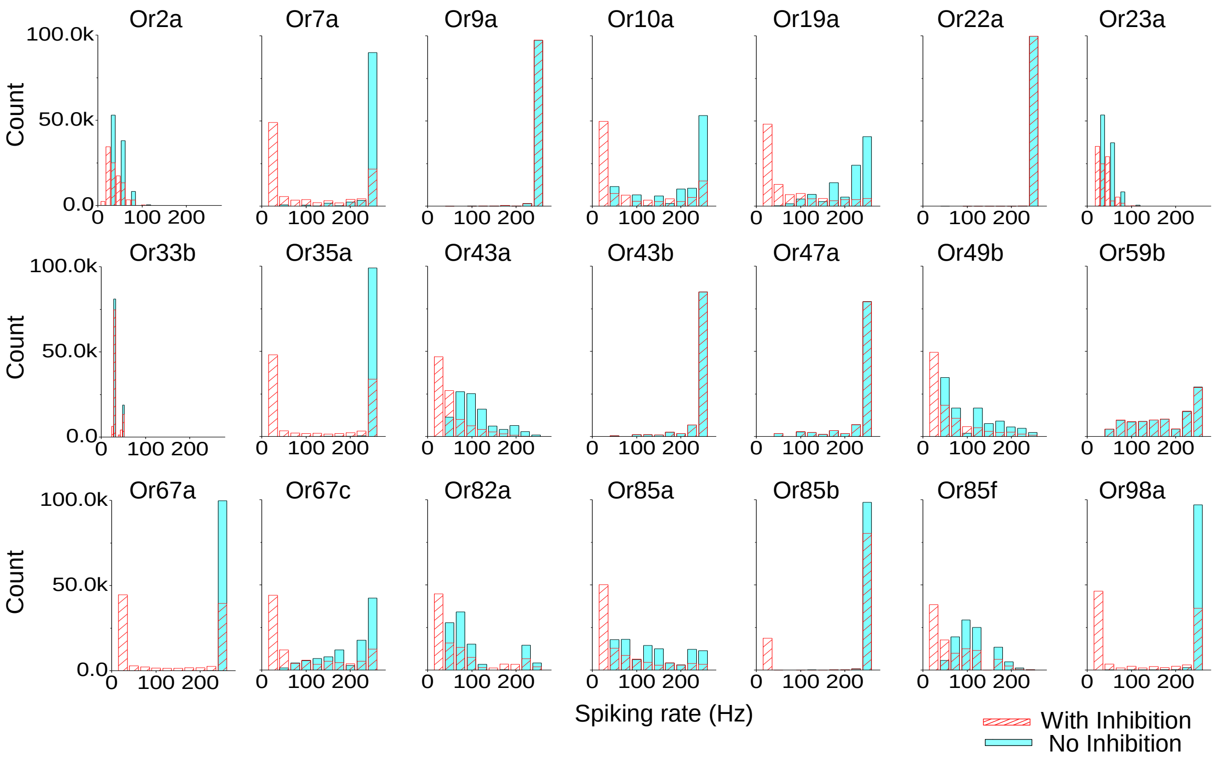

Supplementary Figure 8

Decorrelation of odor responses by inhibition

Including the inhibitory response avoids saturation and makes the response more uniform. The number of odors in the mixture was 20. The sample size was 100,000 odor mixtures.

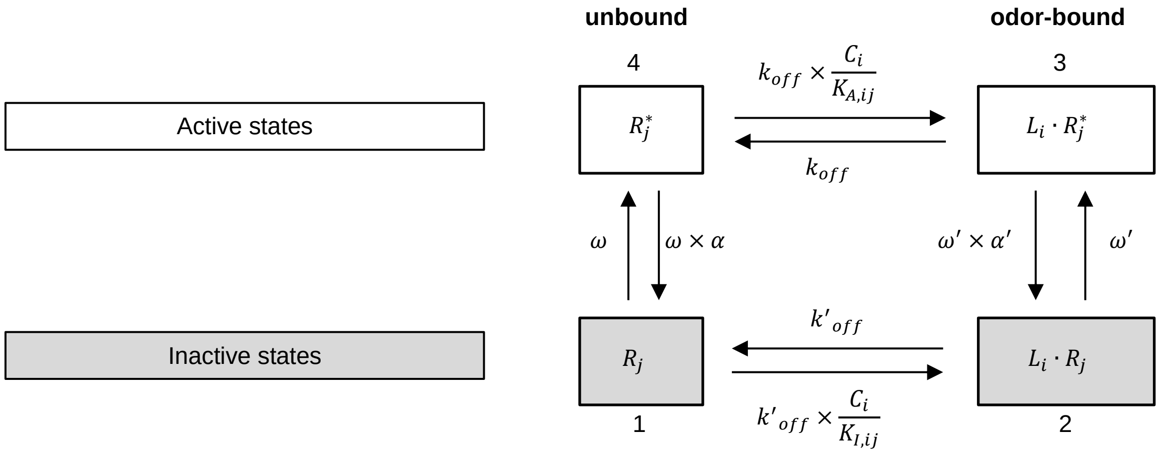

Supplementary Figure 9

Illustration of the different microscopic states of ORs

The active and inactive forms of the OR/OSN$_j$ are represented by $R^*{}_j$ and R$_j$ respectively. The transition rates are given next to the corresponding arrows. The odor is represented by L_i, k_{off}, k'_{off}, ω, ω′are kinetic rates, α′ is a ratio of kinetic rates.

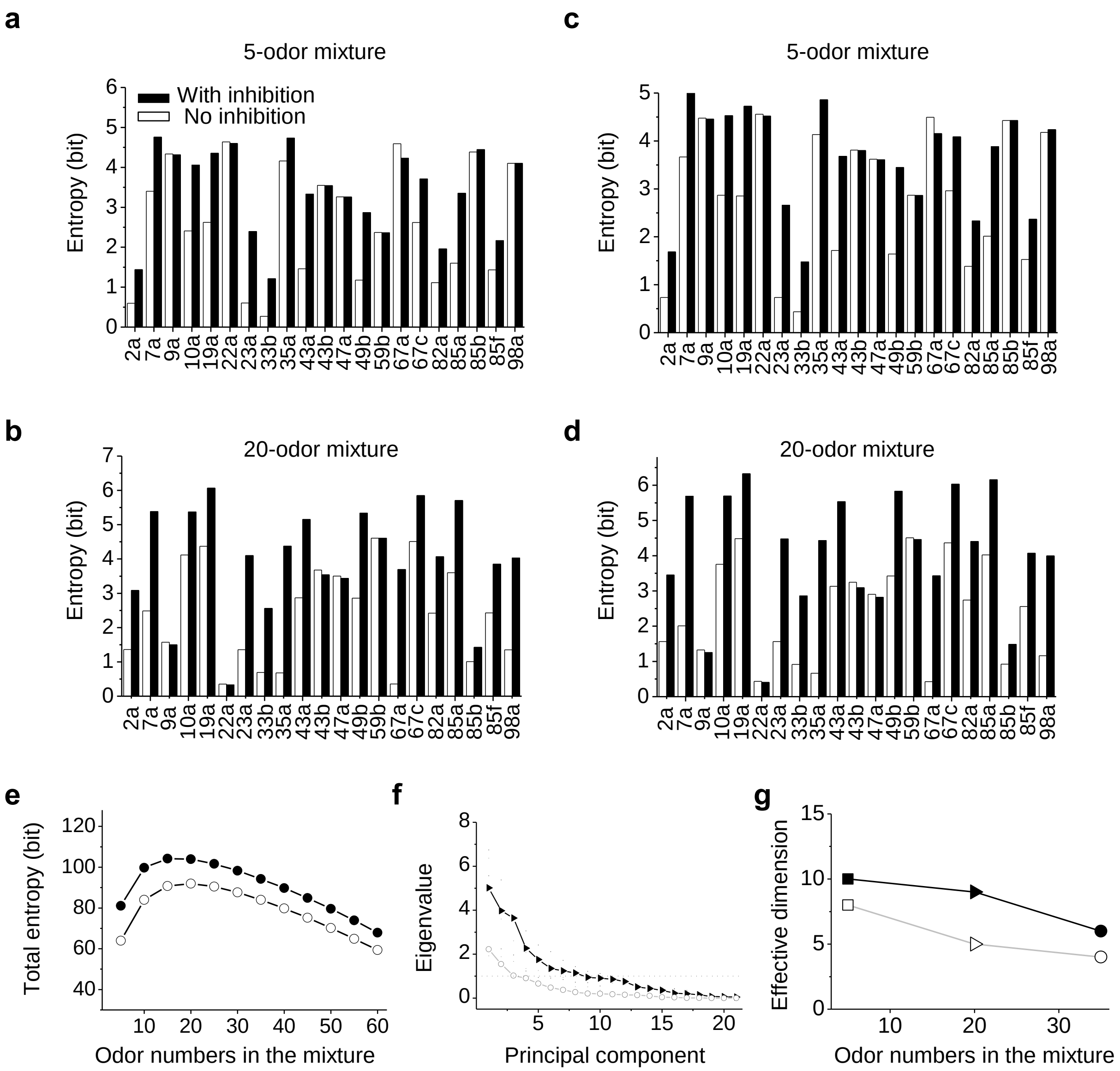

Supplementary Figure 10

Including inhibition increased the information entropy of each OR/OSN

(**a**) The entropy was computed for 100,000 randomly sampled mixtures containing 5 odors. (**b**) The entropy was computed for 100,000 randomly sampled mixtures containing 20 odors. (**c**) The entropy was computed with the enumeration method for mixtures containing 5 odors. (**d**) The entropy was computed with the enumeration method for mixtures containing 20 odors. (**e**) The entropy was calculated for mixtures containing different numbers of odors with competitive binding using the enumeration method. Including inhibition increased the total entropy. (**f**) The eigenvalues of principal components increase by including inhibition. The mixture contained 20 odors. (**g**) The effective coding dimension, defined as the number of principal components whose eigenvalues are larger than 1 (dashed horizontal line in **f**).

Supplementary Table 1

Properties of acetophenone-evoked inhibition of basal activities and E-3-evoked excitatory responses in *Or85a*-OSNs

Odor stimuli	R_{max} (pA)	t_{peak} (ms)	$K_{1/2}$ (mM)
Acetophenone	18.2 ± 2.2 (n = 42)	275 ± 25 (n = 4)	35 ± 11 (n = 4)
E-3	92.6 ± 24.4 (n = 5)	192 ± 26 (n = 5)	1.1 ± 0.1 (n = 5)
E-3 (in the presence of 7.5 mM acetophenone)	92.8 ± 24.6 (n = 5)	192 ± 26 (n = 5)	26.8 ± 12.5 (n = 5)

t_{peak}, or time to peak, is defined as the time duration from odor arrival to the transient peak of odor-evoked responses.
